# Real-world estimation taps into basic numeric abilities

**DOI:** 10.3758/s13423-024-02575-4

**Published:** 2024-10-28

**Authors:** Barbara K. Kreis, Julia Groß, Thorsten Pachur

**Affiliations:** 1 Department of Psychology, University of Mannheim, Mannheim, Germany; 2 School of Management, Technical University of Munich, Munich, Germany; 3 Center for Adaptive Rationality, Max Planck Institute for Human Development, Berlin, Germany

**Keywords:** Real-world estimation, Symbolic-number mapping, Domain knowledge, Number-line task

## Abstract

Accurately estimating and assessing real-world quantities (e.g., how long it will take to get to the train station; the calorie content of a meal) is a central skill for adaptive cognition. To date, theoretical and empirical work on the mental resources recruited by real-world estimation has focused primarily on the role of domain knowledge (e.g., knowledge of the metric and distributional properties of objects in a domain). Here we examined the role of basic numeric abilities – specifically, symbolic-number mapping – in real-world estimation. In Experiment 1 ($N=286$) and Experiment 2 ($N=592$), participants first completed a country-population estimation task (a task domain commonly used to study real-world estimation) and then completed a number-line task (an approach commonly used to measure symbolic-number mapping). In both experiments, participants with better performance in the number-line task made more accurate estimates in the estimation task. Moreover, Experiment 2 showed that performance in the number-line task predicts estimation accuracy independently of domain knowledge. Further, in Experiment 2 the association between estimation accuracy and symbolic-number mapping did not depend on whether the number-line task involved small numbers (up to 1000) or large numbers that matched the range of the numbers in the estimation task (up to 100,000,000). Our results show for the first time that basic numeric abilities contribute to the estimation of real-world quantities. We discuss implications for theories of real-world estimation and for interventions aiming to improve people’s ability to estimate real-world quantities.

## Introduction

People commonly need to estimate unknown quantities in their daily lives – whether gauging how long it will take to get to the train station or assessing the calorie content of a meal. Correctly understanding and estimating such quantities can be highly relevant: It is important not to miss one’s train, and to be able to evaluate whether one’s calorie intake is in line with one’s nutritional goals. Which mental resources are involved in the estimation of real-world quantities?

To date, theoretical and empirical work on real-world estimation – which has been studied across various domains, including country populations, city-to-city distances, longitudes and latitudes, sugar content of food items, frequency of health risks, the number of people participating in different sports, and tuition fees for U.S. universities (Brown & Siegler, 1993, 1996, 2001; Friedman & Brown, 2000; Groß et al., 2024; Lawson & Bhagat, 2002; Pachur, 2024; Pachur et al., 2013) – has focused primarily on the role of domain knowledge. Domain knowledge refers to any knowledge that a person might have of a given domain (e.g., country populations), including knowledge of both qualitative aspects (e.g., geographical features that indicate uninhabitable land such as deserts) and quantitative aspects (e.g., exemplars such as the population of a specific country, or ordinal relationships between countries; Brown, 2002; Brown & Siegler, 1993; Lawson & Bhagat, 2002).

However, is domain knowledge the only mental resource contributing to the accurate estimation of real-world quantities? The role of more basic numeric abilities, such as symbolic-number mapping, has received considerable attention in the context of laboratory tasks involving quantities (e.g., memory for numbers or preference for monetary lotteries) but has not yet been considered in the context of real-world estimation. Symbolic-number mapping (Schneider et al., 2018; Thompson & Siegler, 2010; Peters & Bjalkebring, 2015; Schley & Peters, 2014) refers to a person’s ability to correctly represent magnitudes proportionally to each other on a mental number line. It is often measured with the number-line task (Siegler & Opfer, 2003), where participants are presented with a blank horizontal line marked only with a range (usually from 0 to 100 or 1000) and asked to map a given number (e.g., 42) onto the line (Schneider et al., 2018; Siegler & Opfer, 2003). Performance in the number-line task develops throughout childhood and shifts from a logarithmic to a more linear mapping (Siegler et al., 2009; Opfer & Siegler, 2007). Symbolic-number mapping has been shown to be associated with performance in various complex numeric tasks. For instance, more accurate symbolic-number mapping is related to a better memory for numbers (Thompson & Siegler, 2010; Peters & Bjalkebring, 2015), to choosing the normatively better option more frequently in a risky choice task (Peters & Bjalkebring, 2015; Patalano et al., 2020), and to trading off money and time more proportionally (Schley & Peters, 2014).

Might symbolic-number mapping also be involved in real-world estimation? To date, the two strands of research have existed independently. Studies on the relationship between symbolic-number mapping and judgment and decision-making have, for the most part, relied on decision-making paradigms in which participants are presented with experimentally designed numeric stimuli (e.g., lotteries, numbers of objects; Patalano et al., 2020; Peters & Bjalkebring, 2015; Schley & Peters, 2014). All the information needed to solve the task is provided by the experimenter. By contrast, research on the estimation of real-world quantities relies on real-world stimuli. Here, participants have to retrieve relevant information learned outside the lab from memory. They generate an estimate by integrating various pieces of numeric and non-numeric information from their real-world knowledge. Due to these profound differences in stimuli and task requirements, it seems difficult to determine whether symbolic-number mapping may also play a role in real-world estimation.

However, on a procedural level, there are indications this might be the case. As described by Brown (2002), a real-world estimate often has to be constructed based on a ballpark notion of the general metric of the objects in a domain, and on assessments of the ordinal position of the objects. Specifically, once a general metric or response range for objects in a domain has been set, “estimates are generated by determining the relative or ordinal value of the target item and selecting a numerical value from the appropriate portion of the range” (p. 326). Arguably, mapping the position of objects onto a metric range recruits abilities similar to those required in symbolic-number mapping tasks (even though symbolic-number mapping, unlike real-world estimation, also requires perceptual skills). However, it has not yet been tested whether the accuracy of real-world estimation is associated with symbolic-number mapping.

If such an association exists, this would be informative for theories on real-world estimation as well as for interventions aiming to improve people’s ability to estimate real-world quantities. Specifically, in addition to domain-specific approaches such as conveying quantitative knowledge of a domain (e.g., the calorie content of different food items), interventions could include domain-general approaches that target basic numeric abilities, such as providing feedback on the number-line task (e.g., Fitzsimmons et al., 2023; Opfer & Siegler, 2007; Thompson & Opfer, 2016).

Our goal in this article is to assess whether the accuracy of real-world estimation is associated with symbolic-number mapping. In two experiments, participants estimated the population of various countries (a domain commonly used to investigate real-world estimation; e.g., Brown, 2002; Brown & Siegler, 1993, 1996) and performed a number-line task. Experiment 2 further sought to disentangle the effects of symbolic-number mapping and domain knowledge by examining whether performance in the number-line task predicts estimation accuracy independently of domain knowledge; and tested whether the strength of the association between estimation accuracy and symbolic-number mapping depends on the match between the range of the quantities to be estimated and the range of the numbers to be mapped.

**Fig. 1:**
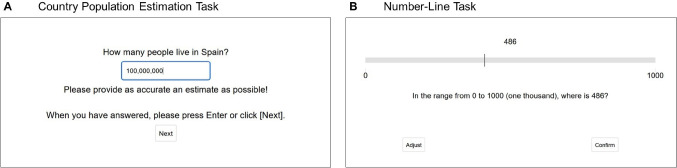
Sample item from (**A**) the estimation task and (**B**) the number-line task. Translated from German

## Experiment 1

### Method

Experiment 1 was part of a larger study investigating the role of knowledge updating in hindsight bias (Groß et al., 2023, Experiment 2). In a first phase, participants estimated the population of several countries. In a second phase, they received different types of numeric information, depending on the experimental condition to which they were assigned. In a third phase, participants were asked to recall their responses from the initial estimation phase. In a fourth phase, they provided estimates for a new set of countries. In a fifth and final phase, participants completed a number-line task. For the present purposes, only the initial estimation phase and the number-line task are relevant. A more comprehensive description of the full design and results can be found in Groß et al. (2023), Experiment 2. The experiment was not preregistered.

#### Participants

A sample of 295 participants was recruited via Prolific (www.prolific.co). All participants were native speakers of German, aged 18 to 45 years ($M= 27.8$ years, 116 women, 178 men, one diverse). The majority of participants were currently working (130) or studying (university or college, 122), 29 participants were looking for work, seven were high-school students, six were apprentices, and one was retired. The median completion time for the entire experiment was 26.4 min and participants were paid a fixed compensation of £5.60.

#### Material

For the estimation task, we compiled three sets of items consisting of 32 countries each (see Appendix Table 1). Each participant estimated one item set. The sets were assigned to participants such that they were presented with comparable frequency across participants. In the number-line task, participants were presented with 22 numbers ranging from 2 to 938, which they were asked to map onto a 20-cm horizontal line, labeled from 0 on the left to 1000 on the right (see Procedure and design for details).

#### Procedure and design

The experiment was programmed with lab.js (Henninger et al., 2022) and hosted via Open lab (Shevchenko, 2022). After providing informed consent and demographic information, participants completed an estimation task, in which they estimated the population of 32 countries (see Fig. 1, panel A, for a sample item). Responses smaller than 1000 were not allowed, as they would likely reflect either a misunderstanding of the task or a lack of attention. The country names were presented sequentially and in random order for each participant. Participants took a median time of 8.1 seconds for each item. Finally, participants completed the number-line task, in which they indicated the position of 22 numbers on a horizontal number line with a mouse click (see Fig. 1, panel B for a sample item). The numbers were presented sequentially and in fixed order (486, 122, 34, 163, 56, 754, 725, 366, 147, 2, 938, 606, 78, 818, 246, 722, 18, 738, 150, 5, 179, 100; see Opfer & Siegler, 2007). Responses could be corrected if necessary. Participants took a median time of 5.4 s for each number. At the end of the experiment, participants could provide comments of any kind in an open response field.

#### Data diagnostics

Several exclusion criteria were applied to ensure good data quality. First, participants were automatically excluded by Prolific if they did not finish the study within a specified time limit (106 min for a study with an estimated finishing time of 40 min). Second, we excluded participants who reported technical problems (two participants), having been considerably distracted during participation (one participant), or having looked up actual population figures during the estimation task (one participant). Third, we excluded all responses that were equal to or larger than the current world population, 8 billion, and thus unrealistic (8 responses, $0.09\%$ of all responses in the estimation task). Fourth, we checked for extremely fast responses (i.e., below 1 s), but there were no such responses for either task. Fifth, we excluded participants whose median order of magnitude error (our measure of estimation accuracy; see Eq. 1 below) in the estimation task exceeded the threefold interquartile range (five participants). Sixth, we excluded responses in the number-line task that exceeded the threefold interquartile range for a given number (42 responses, $0.65\%$ of all responses in the number-line task). Finally, we checked whether more than 20% of number-line task responses were excluded for any given participant; this was not the case. The exclusions resulted in a final sample of 286 participants. The data and the analysis code are available at https://osf.io/34dvr/.

#### Analytic approach

To quantify accuracy in the estimation task, we calculated the deviation of the estimated country population from the actual country population in terms of the order of magnitude error (OME) for each item *i* and for each participant *j* (Brown & Siegler, 1996):

1 $$\begin{aligned} OME_{ij} =|log_{10}\left( \frac{estimate_{ij}}{actual_i}\right) |. \end{aligned}$$

The OME is a function of the difference between the estimated and the actual value of an item and converts the difference into an order of magnitude. A larger OME indicates a larger error, that is, lower estimation accuracy. The OME has been frequently used in the context of country-population estimation.^1^

To quantify accuracy in the number-line task, we calculated the relative deviation of the number indicated on the line from the target number for each item *k* and each participant *j*:

2 $$\begin{aligned} \Delta _k{_j} = |\left( \frac{estimate_k{_j} - actual_k}{actual_k}\right) |. \end{aligned}$$

This measure is commonly used to quantify accuracy in the number-line task, with larger values indicating lower accuracy (Schneider et al., 2018). Each participant’s accuracy in the number-line task is calculated as the median $\Delta$ across items. A larger $\Delta$ indicates lower symbolic-number mapping accuracy.

We used Bayesian linear mixed-effects regression modeling to test whether estimation accuracy (operationalized as the OME) was associated with symbolic-number mapping (operationalized as median $\Delta$). Specifically, the model predicted the OME of participant *j* for each item *i* of the estimation task, using as fixed effect the participant’s median $\Delta$. The model further included random intercepts for participants and items to take by-person and by-item variability in estimation accuracy into account. The analyses were conducted with the brms package (Bürkner, 2017, 2018), which calls STAN for MCMC sampling (Stan Development Team, 2019). Prior specification and sensitivity analyses are provided in Appendix B. The general conclusions were robust across different prior specifications.

To statistically evaluate the effects, we compared the full model including a given fixed effect (i.e., symbolic-number mapping), *M*_1_, to the baseline model without that effect, *M*_0_. The baseline model included all random effects that were specified in the full model. We compared the models using the bayes_factor function in brms, which computes Bayes factors (BF) based on bridge sampling (e.g., Gronau et al. 2017). The BF_10_ quantifies the evidence for the alternative hypothesis relative to the null hypothesis by comparing the full model *M*_1_ to the baseline model *M*_0_.^2^

### Results

**Fig. 2:**
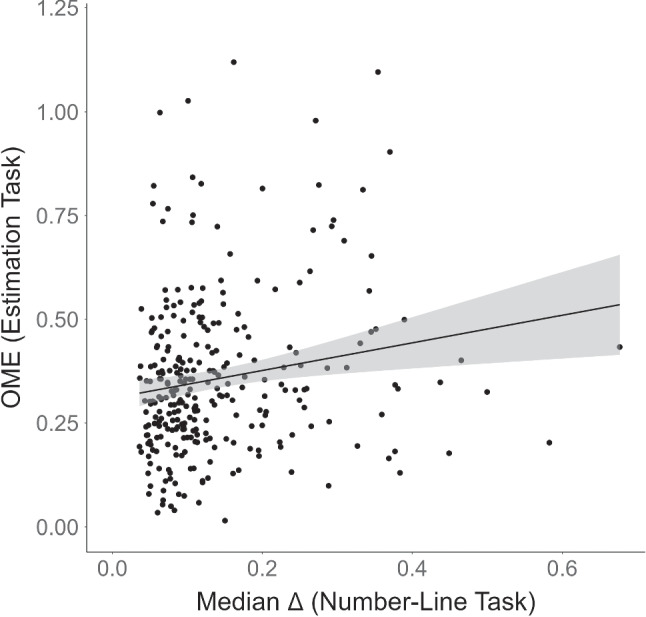
Association between performance in the estimation task and performance in the number-line task in Experiment 1. OME = order of mag-nitude error. Each point represents the performance of a participant. For the OME, the median (across items) for each participant is shown. For both OME and median $\Delta$, larger values indicate worse performance. The uncertainty band around the regression line represents the standard error

Participants’ average estimation accuracy, measured in terms of median OME across items, was $M = 0.36$ ($SD = 0.19$; range 0.02–1.12). Participants’ average symbolic-number mapping accuracy, measured in terms of median $\Delta$, was $M = 0.14$ ($SD = 0.1$; range 0.04–0.68). As Fig. 2 shows, symbolic-number mapping accuracy was associated with accuracy in the estimation task. The results of the mixed-effects model indicated that participants who were more accurate in symbolic-number mapping also provided more accurate estimates for the country populations ($b = 0.38$, $CI_{95\%}$ = [0.16, 0.59]).^3^

The standardized regression weight was $\beta = 0.09$, implying that an increase of one standard deviation in symbolic-number mapping accuracy was associated with an increase of 0.09 standard deviations in estimation accuracy. Although the size of the effect may appear modest, evidence for it was strong (BF_10_ $= 83$).^4^

### Discussion

Experiment 1 suggests that basic numeric abilities contribute to real-world estimation. Given the novelty of this finding, replicating it seems desirable. Furthermore, although the statistical evidence for the association was strong, the size of the effect was rather modest. One possible reason is that the number range of the number-line task (up to 1000) did not match that of the estimation task (where the country populations ranged in the millions). The effect size may be larger if the two ranges match. To address this possibility, in Experiment 2 we included two number-line task conditions, one with small (up to 1000) and one with large (up to 100,000,000) quantities. Finally, individuals with a higher symbolic number-mapping ability might also have more knowledge about the domain (e.g., due to a common link with general intelligence). The association observed between symbolic-number mapping and estimation accuracy could thus be due to a confound. We addressed this possibility in Experiment 2 by including a measure of domain knowledge, allowing us to statistically control for domain knowledge.^5^

## Experiment 2

### Method

The experiment was preregistered (see https://aspredicted.org/25K_F7H).

#### Participants

To determine the target sample size, we conducted a simulation-based a priori power analysis based on the data from Experiment 1 with the mixedpower package in R (Kumle et al., 2021). We simulated power for the full model that included symbolic-number mapping (operationalized as median $\Delta$) as a fixed effect. We ran 500 simulations, and defined a critical *t* value of 2, corresponding to an $\alpha$ level of $5\%$. The simulation showed that a power of $95\%$ would be achieved with 290 participants. To ensure that our sample size would be sufficient for both individual and joint analyses of the two number-line conditions, we doubled this figure to 580. To account for potential exclusions, we recruited an additional 60 participants, resulting in an overall sample of $N=640$.

Participants were only allowed to take part in the experiment if they had not taken part in Experiment 1. Of the $N=640$ participants who submitted their data on Prolific (www.prolific.co), $N=636$ completed the experiment. The median completion time was 16.7 min and participants received a fixed compensation of £3.70. All participants were native speakers of German, aged 18 to 45 years ($M= 27.9$ years, 277 women, 346 men, 10 diverse, 3 not specified). The majority of participants were working (318) or studying (university or college, 239), 46 were unemployed (33 looking for work, 13 not looking for work), 11 were high-school students, and 22 were apprentices.

#### Material and design

The estimation task included a fixed set of 46 countries (see Appendix Table 2). We increased the number of items relative to Experiment 1 (which included 32 countries) to increase the power and reliability of the measure. Participants were randomly assigned to one of two conditions of the number-line task. In the *Thousand* condition the number line ranged from 0 to 1000. The task was identical to the number-line task in Experiment 1, with the exception that 18 further numbers were added to achieve a more even distribution across the number range; participants thus mapped a total of 40 numbers (see Procedure for details). In the *Millions* condition the number line ranged from 0 to 100,000,000 and involved the same 40 numbers as in the *Thousand* condition but multiplied by 100,000.

We measured domain knowledge of country populations with a *Domain Engagement Question*. Participants were asked to indicate how often they had engaged with the topic of country populations prior to this study on a seven-point scale. Response options ranged from “very rarely” to “very frequently” (see Appendix  D for details). We opted for this approach to measure domain knowledge for several reasons. Asking participants to rate their knowledge directly (Ainley et al., 2002) might tap into self-assessment accuracy and past success at estimating country populations, the latter likely being influenced by a mixture of basic numeric abilities and prior knowledge. Our more indirect approach circumvents this potential problem, focusing on the frequency of engagement with the topic as a purer indicator of the amount of knowledge acquired (e.g., through education and the media).

#### Procedure

The experiment was programmed with lab.js (Henninger et al. 2022) and hosted via Jatos (Lange et al., 2015). After providing informed consent, participants were asked to estimate the population of 46 countries, one at a time. As in Experiment 1, responses smaller than 1000 were not allowed. Participants took a median time of 7.2 s for each item. Unlike in Experiment 1, the country names were presented in fixed order for all participants. Subsequently, participants performed the number-line task. The procedure and the instructions were the same as in Experiment 1. All 40 numbers were presented sequentially in a fixed order (for the *Thousand* condition: 486, 319, 651, 547, 5, 100, 214, 18, 573, 827, 385, 905, 163, 179, 147, 302, 56, 863, 122, 2, 534, 439, 722, 983, 366, 738, 597, 725, 685, 246, 150, 78, 291, 818, 754, 872, 34, 938, 413, 606). The median response times were 4.3 s and 4.4 s in the *Thousand* and *Millions* conditions, respectively. After completing the two tasks, participants answered the Domain Engagement Question, provided demographic information, and indicated whether they had participated seriously and whether they had cheated (i.e., looked up answers, and/or asked others for help). Finally, participants could provide comments of any kind in an open response field.

#### Data diagnostics

We preregistered several exclusion criteria. First, participants were automatically excluded by Prolific if they did not finish the study within a specified time limit (71 min for a study with an estimated finishing time of 22 min). For one participant, this exclusion did not work due to technical reasons. The participant had an overall completion time of over 4 h, and was manually excluded by us. Second, we excluded participants who reported problems with the experiment (one participant), having cheated (14 participants), or having just clicked through (three participants). Third, we excluded all responses in the estimation task that were equal to or larger than 8 billion (43 trials, 0.15% of responses in the estimation task). Fourth, we excluded participants whose responses took less than 1 s for more than 10% of items (in both tasks); no such cases occurred. Fifth, we excluded participants whose median OME in the estimation task exceeded the threefold interquartile range (19 participants). Sixth, we excluded responses in the number-line task that exceeded the threefold interquartile range for a given number (separately for the *Thousand* and *Millions* condition); overall, 315 responses were excluded (1.24%). Finally, we excluded participants for whom more than 20% of the responses in the number-line task had to be excluded; this was the case for six participants. The final sample consisted of $N=592$ participants, with $n=303$ participants in the *Thousand* condition and $n=289$ participants in the *Millions* condition.

#### Analytic approach

As in Experiment 1, we quantified accuracy in the estimation task as the OME (Eq. 1) and accuracy in the number-line task as the median $\Delta$ (Eq. 2). To quantify domain knowledge, we converted the responses on the Domain Engagement Question to numbers ranging from 1 (for *very rarely*) to 7 (for *very frequently*).

We used a Bayesian linear mixed-effects regression approach to test for associations of estimation accuracy with symbolic-number mapping and domain knowledge. Specifically, the models predicted the OME of participant *j* for each item *i* of the estimation task. As fixed effects, we used the median $\Delta$ of participant *j*, the number-line task condition they were assigned to (*Thousand* vs. *Millions*), as well as their response to the Domain Engagement Question. In addition to the fixed effects, all models included random intercepts for participants and items to take by-person and by-item variability in estimation accuracy into account. Prior specification and sensitivity analyses are described in Appendix B. Again, the general conclusions were robust across different prior specifications. The data and the analysis code are available at https://osf.io/34dvr/.

### Results

#### Is estimation accuracy associated with symbolic-number mapping?

**Fig. 3:**
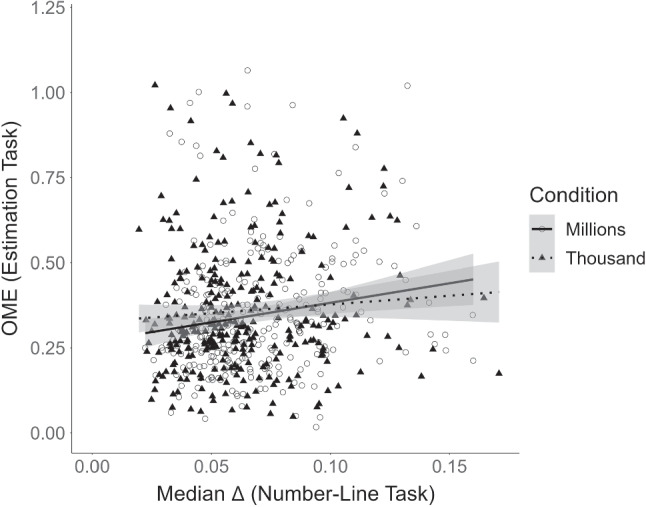
Association between performance in the estimation task and performance in the number-line task in Experiment 2 by number-line condition. OME = order of magnitude error. Each *point* represents the performance of a participant. For the OME, the median (across items) for each participant is shown. For both OME and median $\Delta$, larger values indicate worse performance. The uncertainty band around the regression line represents the standard error

Participants’ accuracy in the estimation task was comparable to that of Experiment 1, with a median (across items) OME of $M = 0.35$ ($SD = 0.19$; range 0.02–1.07), on average. Accuracy in the number-line task measured in terms of median $\Delta$, was $M = 0.07$ ($SD = 0.03$; range 0.02–0.17), on average, and thus better than in Experiment 1 ($M = 0.14$, $SD = 0.1$, range 0.04–0.68). Nevertheless, as Fig. 3 shows, estimation accuracy and accuracy in symbolic-number mapping were again positively associated, as in Experiment 1. Across both number-line conditions, participants with more accurate symbolic-number mapping also did better in the estimation task. This association was corroborated by a mixed-effects model predicting estimation accuracy from symbolic-number mapping ($b = 0.70$, $CI_{95\%}$ = [0.18, 1.21], BF_10_ $= 34$). The standardized regression weight was $\beta = 0.04$.

#### Does the association depend on the number range used to measure symbolic-number mapping?

Accuracy in the estimation task was similar across the two number-line conditions, with a median (across items) OME of $M = 0.36$ ($SD =0.19$; range 0.05–1.02), on average, in the *Thousand* condition and $M = 0.35$ ($SD = 0.18$; range 0.02–1.07), on average, in the *Millions* condition. The same held for accuracy in the number-line task, with $M = 0.06$ ($SD = 0.03$; range 0.02–0.17) in the *Thousand* condition and $M = 0.07$ ($SD = 0.03$; range 0.02–0.16) in the *Millions* condition. To test whether the size of the association was larger in the *Millions* condition than in the *Thousand* condition, we compared a model including the interaction of symbolic-number mapping and number-line condition with a baseline model containing only the two fixed effects. Results showed that the association did not differ between the two conditions ($b = -0.03$, $CI_{95\%}$ = [$-0.75, 0.69$]). While the credible interval of the regression coefficient of the interaction term included zero, the Bayes factor of BF_10_ $= 0.62$ indicated only weak evidence for the absence of an interaction.

#### Does symbolic-number mapping predict estimation accuracy beyond domain knowledge?

Participants’ response on the Domain Engagement Question (ranging from 1 = very rarely to 7 = very frequently), was $$M = 2.58$$ ($$SD = 1.37$$), on average. Domain knowledge was uncorrelated with symbolic-number mapping ($$r =.00$$, BF_10_ = 0.10), but it was associated with estimation accuracy ($$b = -0.05$$, $$CI_{95\%}$$ = [$$-0.07, -0.04$$], BF_10_ $$> 100,000$$). The standardized regression coefficient was $$\beta = -0.17$$, indicating that an increase of one standard deviation in domain knowledge was associated with an increase of 0.17 standard deviations in estimation accuracy (i.e., a decrease in OME). To test whether estimation accuracy was associated with symbolic-number mapping when statistically controlling for domain knowledge, we compared a model including both domain knowledge and symbolic-number mapping as fixed effects to a baseline model that included only domain knowledge as a fixed effect. Symbolic-number mapping predicted estimation accuracy even when domain knowledge was included as a covariate ($$b = 0.71$$, $$CI_{95\%}$$ = [0.24, 1.20], BF_10_ $$ = 30$$). The standardized regression coefficient of symbolic-number mapping was $$\beta = 0.04$$.^6^

### Discussion

We replicated the key finding of Experiment 1, namely, that symbolic-number mapping is associated with accuracy in real-world estimation. The predictive strength of symbolic-number mapping was smaller in Experiment 2 ($\beta = 0.04$) than in Experiment 1 ($\beta = 0.09$). This could be due to the overall higher accuracy (i.e., lower median $\Delta$) and smaller variability in symbolic-number mapping in Experiment 2 ($M = 0.07$, $SD = 0.03$, range 0.02–0.17; Experiment 1: $M = 0.14$, $SD = 0.1$, range 0.04–0.68). As Experiment 2 was shorter than Experiment 1, the higher accuracy may be due to higher participant motivation or concentration. Importantly, however, symbolic-number mapping emerged as a robust predictor of real-world estimation accuracy in both experiments, irrespective of differences in accuracy and variability.

Furthermore, the strength of the association between symbolic-number mapping and real-world estimation was not dependent on whether symbolic-number mapping was measured using numbers that matched the range of quantities in the estimation task (country populations in the millions) or not. This indicates that the facet of symbolic-number mapping that is linked to real-world estimation is independent of the range of the numbers used to measure symbolic-number mapping.

Finally, our findings underscore that domain knowledge plays an important role for accurate real-world estimation (Brown & Siegler, 1993; Brown, 2002): Self-reported domain knowledge was a stronger predictor of estimation accuracy ($\beta = -0.17$) than was symbolic-number mapping ($\beta = 0.04$). Crucially, however, symbolic-number mapping predicted estimation accuracy beyond domain knowledge, suggesting a unique role of symbolic-number mapping.

## General discussion

Accurately estimating real-world quantities is a relevant skill in daily life. However, empirical and theoretical work on the mental resources that contribute to real-world estimation is scarce. While domain knowledge has been shown to play a role (Brown & Siegler, 1993; Brown, 2002), the potential contribution of basic numeric abilities has not yet been considered. The two experiments reported in this article demonstrated that real-world estimation is reliably associated with symbolic-number mapping. Importantly, we showed that this association is not merely an epiphenomenon arising from a confound between symbolic-number mapping and domain knowledge (e.g., via intelligence): Domain knowledge and basic numeric abilities seem to contribute independently to real-world estimation.

### Theoretical implications

Our results can inform and further specify theoretical ideas on real-world estimation. Brown (2002) proposed a conceptual framework outlining the processes that people may engage in when estimating real-world quantities. According to this proposal, people first come up with a general idea of the metric magnitude of the domain in question, such as the range or distribution of objects in that domain. They then locate the relative position of the object to be estimated in this metric range. Where in this process could basic numeric abilities come into play? One possibility is that they influence the estimation process itself: More accurate symbolic-number mapping might allow people to define a more appropriate metric range and to pinpoint the relative position of the object more accurately. Symbolic-number mapping could also facilitate the development of a relevant knowledge base that is recruited for real-world estimation. When presented with numeric information about a domain, people with more accurate symbolic-number mapping might be better able to integrate this information into existing knowledge. Consistent with this idea, people with better symbolic-number mapping have been shown to perform better in memory tasks involving numbers (Thompson & Siegler, 2010; Peters & Bjalkebring, 2015).

### Practical implications

Our insights into the mental resources contributing to real-world estimation can inform the development of interventions to boost citizens’ ability to estimate real-world quantities. A common approach here is to improve people’s domain knowledge by presenting them with a representative selection of items from the corresponding domain (Bröder et al., 2023; Brown & Siegler, 1993; Groß et al., 2024; Marghetis et al., 2019). Although this kind of intervention is simple and effective, the expected improvements are necessarily limited to the specific domain.

Our findings suggest that interventions to boost real-world estimation could also benefit from a more domain-general approach that aims to improve symbolic-number mapping. This could be achieved, for instance, by providing people with corrective feedback on the placement of numbers in a number-line task, or by presenting them with worked examples and asking them to explain why they are correct or incorrect. Prior research suggests that such interventions can improve accuracy not only for symbolic-number mapping itself, but also for other numeric tasks such as memory for numbers (Opfer & Siegler, 2007; Thompson & Opfer, 2016; Fitzsimmons et al., 2023). Improving symbolic-number mapping could improve estimation accuracy by helping people to reorganize their existing knowledge base and correct inaccurate relational ordering or mapping of objects in a domain. In addition, such interventions might help people to integrate numerical information more accurately into the knowledge base, making subsequent retrieval and use of that information more effective.

### Limitations and outlook

In Experiment 2, participants’ performance in the number-line task was not affected by whether it involved small or large numbers. This result may seem surprising, given that prior studies reported poorer number-line performance for large numbers (e.g., Landy et al., 2013, 2017). A possible explanation for the similar accuracy in the *Thousand* and *Millions* conditions is that the participants simplified the *Millions* task by mentally crossing out the zeros at the end of the numbers. Future studies could employ numbers without zeros at the end to see whether this affects the results. Note, however, that Landy et al. (2013) and Landy et al. (2017) also used large numbers with zeros at the end and still observed lower mapping accuracy for larger than for smaller numbers. Another possible reason that Landy and colleagues (Landy et al., 2013, 2017) found lower number-line performance for larger than for smaller numbers, whereas we did not, could be that our number-line task with larger numbers only ranged up to 100 million (to match the country populations), whereas it ranged up to a billion in Landy et al. (2013) and Landy et al. (2017).

A potential limitation of our approach to measuring domain knowledge in Experiment 2 is that we used a single self-report item: Participants indicated how often they had engaged with the topic of country populations prior to our study. Arguably, this is a rather indirect measure of domain knowledge. Future studies might consider using genuine knowledge questions about the domain, as in Light et al. (2022). Additionally, future work could include a general measure of cognitive ability, such as IQ, to further clarify the relationship of estimation accuracy with symbolic-number mapping and domain knowledge.

Further, given that our estimation task focused on a specific (though commonly investigated) real-world domain – country populations – it is an open question to what extent our conclusions generalize to other knowledge domains. In general, the relative contribution of symbolic-number mapping and domain knowledge is likely to vary depending on the amount of knowledge available in a domain. For instance, basic numeric abilities might play a smaller role for estimating quantities in familiar domains, such as the sugar content of food items, than in less familiar domains, such as the carbon footprint of food items (Bröder et al., 2023; Groß et al., 2024). Overall, however, we have no basis to assume that the role of basic numeric abilities will disappear completely in familiar domains.

Furthermore, there is a discrepancy between the estimation task and the number-line task regarding the scale format: the estimation task used an open-ended scale, whereas the number-line task used a bounded scale with a clearly defined endpoint. This reflects how these tasks are usually implemented in the literature (for a measurement of symbolic-number mapping with an open-ended scale, see Reinert & Moeller, 2021). Future research could examine to what extent the association between real-world estimation and symbolic-number mapping is affected when both tasks use the same type of response scale (open-ended or bounded).

### Conclusion

What role do basic numeric abilities such as symbolic-number mapping play in judgment and decision-making? To date, research has focused primarily on decision-making tasks such as monetary lotteries or trade-offs between money and time, where the numeric stimuli are stated explicitly. Here we found evidence that symbolic-number mapping is also involved in a judgment task where a numeric response has to be constructed by integrating both numeric and non-numeric information from complex real-world knowledge. This highlights the potentially general importance of numeric abilities for judgments requiring numeric responses.

## Data Availability

The material for the country-population estimation tasks is provided in Appendix A; the data and analysis code are available via the Open Science Framework https://osf.io/34dvr/.

## Appendix A: Materials

Materials are shown in Table 1 (Exp. 1) and Table 2 (Exp. 2).

**Table 1:** Sets of country populations used in Experiment 1

Set 1			Set 2			Set 3		
**1**	203, 177, 034	Pakistan	**1**	211, 819, 321	Brazil	**1**	268, 501, 680	Indonesia
**2**	199, 045, 324	Nigeria	**2**	167, 422, 187	Bangladesh	**2**	143, 919, 453	Russia
**3**	131, 738, 729	Mexico	**3**	107, 505, 862	Philippines	**3**	126, 976, 591	Japan
**4**	97, 074, 662	Vietnam	**4**	100, 488, 879	Egypt	**4**	109, 159, 044	Ethiopia
**5**	85, 705, 256	Democratic Republic of the Congo	**5**	82, 592, 416	Turkey	**5**	82, 518, 959	Iran
**6**	69, 256, 846	Thailand	**6**	59, 246, 609	Italy	**6**	66, 816, 286	United Kingdom
**7**	60, 229, 204	Tanzania	**7**	57, 812, 482	South Africa	**7**	65, 387, 848	France
**8**	51, 273, 440	South Korea	**8**	50, 220, 000	Kenya	**8**	54, 158, 522	Myanmar
**9**	49, 705, 306	Colombia	**9**	46, 439, 538	Spain	**9**	38, 056, 163	Poland
**10**	45, 169, 147	Uganda	**10**	44, 946, 136	Argentina	**10**	37, 156, 729	Canada
**11**	43, 877, 093	Ukraine	**11**	42, 425, 837	Algeria	**11**	36, 468, 117	Morocco
**12**	32, 790, 012	Peru	**12**	40, 002, 380	Iraq	**12**	33, 912, 223	Saudi Arabia
**13**	32, 294, 009	Malaysia	**13**	36, 883, 979	Afghanistan	**13**	32, 642, 668	Uzbekistan
**14**	25, 683, 863	North Korea	**14**	31, 404, 292	Angola	**14**	32, 630, 416	Venezuela
**15**	22, 850, 032	Niger	**15**	29, 858, 634	Ghana	**15**	31, 077, 768	Mozambique
**16**	20, 106, 983	Burkina Faso	**16**	29, 822, 097	Nepal	**16**	25, 295, 354	Ivory Coast
**17**	19, 519, 762	Romania	**17**	29,329,832	Yemen	**17**	20, 992, 622	Sri Lanka
**18**	17, 154, 637	Zimbabwe	**18**	26, 704, 247	Madagascar	**18**	18, 284, 455	Chile
**19**	17, 114, 912	Netherlands	**19**	25, 074, 109	Cameroon	**19**	17, 938, 326	Zambia
**20**	14, 600, 000	Somalia	**20**	24, 970, 495	Australia	**20**	17, 452, 973	Guatemala
**21**	13, 270, 289	Guinea	**21**	19, 510, 631	Malawi	**21**	15, 639, 892	Chad
**22**	11, 683, 042	Benin	**22**	19, 471, 687	Mali	**22**	13, 132, 406	South Sudan
**23**	11, 193, 953	Haiti	**23**	18, 518, 517	Kazakhstan	**23**	11, 980, 000	Rwanda
**24**	11, 133, 944	Greece	**24**	17, 011, 566	Ecuador	**24**	11, 489, 711	Cuba
**25**	10, 629, 078	Czech Republic	**25**	16, 574, 342	Senegal	**25**	11, 443, 124	Burundi
**26**	9, 980, 369	Azerbaijan	**26**	16, 394, 043	Cambodia	**26**	11, 430, 000	Tunesia
**27**	9, 222, 905	Tajikistan	**27**	11, 539, 843	Belgium	**27**	10, 269, 227	Portugal
**28**	8, 582, 983	Switzerland	**28**	11, 318, 180	Bolivia	**28**	10, 026, 898	Sweden
**29**	7, 027, 153	Laos	**29**	10, 953, 914	Dominican Republic	**29**	10, 000, 697	Jordan
**30**	6, 432, 904	El Salvador	**30**	9, 667, 861	Hungary	**30**	9, 439, 781	Belarus
**31**	4, 833, 127	Ireland	**31**	5, 450, 438	Slovakia	**31**	8, 758, 508	Austria
**32**	3, 908, 462	Georgia	**32**	4, 149, 214	Croatia	**32**	7, 006, 598	Bulgaria

**Table 2:** Set of country populations used in Experiment 2

Set			Set (continued)		
**1**	11, 589, 623	Belgium	**24**	31, 072, 940	Ghana
**2**	31, 255, 435	Mozambique	**25**	32, 971, 854	Peru
**3**	60, 461, 826	Italy	**26**	206, 139, 589	Nigeria
**4**	20, 903, 273	Burkina Faso	**27**	69, 799, 978	Thailand
**5**	9, 449, 323	Belarus	**28**	3, 989, 167	Georgia
**6**	102, 334, 404	Egypt	**29**	45, 195, 774	Argentina
**7**	27, 691, 018	Madagascar	**30**	9, 660, 351	Hungary
**8**	29, 825, 964	Yemen	**31**	59, 308, 690	South Africa
**9**	97, 338, 579	Vietnam	**32**	8, 654, 622	Switzerland
**10**	16, 743, 927	Senegal	**33**	51, 269, 185	South Korea
**11**	11, 402, 528	Haiti	**34**	19, 129, 952	Malawi
**12**	67, 886, 011	United Kingdom	**35**	10, 203, 134	Jordan
**13**	11, 673, 021	Bolivia	**36**	10, 847, 910	Dominican Republic
**14**	212, 559, 417	Brazil	**37**	5, 459, 642	Slovakia
**15**	11, 193, 725	South Sudan	**38**	45, 741, 007	Uganda
**16**	109, 581, 078	Philippines	**39**	20, 250, 833	Mali
**17**	40, 222, 493	Iraq	**40**	17, 915, 568	Guatemala
**18**	32, 866, 272	Angola	**41**	17, 643, 054	Ecuador
**19**	43, 851, 044	Algeria	**42**	19, 237, 691	Romania
**20**	4, 105, 267	Croatia	**43**	16, 718, 965	Cambodia
**21**	273, 523, 615	Indonesia	**44**	29, 136, 808	Nepal
**22**	18, 776, 707	Kazakhstan	**45**	84, 339, 067	Turkey
**23**	46, 754, 778	Spain	**46**	38, 928, 346	Afghanistan

## Appendix B: Prior specification and sensitivity analyses

To facilitate specification of the priors, we mean-centered the criterion variable estimation accuracy (operationalized as OME) and the predictors symbolic-number mapping (operationalized as median $\Delta$) and domain knowledge (operationalized as the response to the Domain Engagement Question). We ran prior predictive checks to verify that the priors produced realistic-looking data, as recommended by Schad et al. (2023).

For the intercept parameter (Experiments 1 and 2), we specified a normal distribution normal(0, 1.5). For the standard deviation of the random effects and the residual standard deviation, we specified half-normal distributions, normal(0, 0.5) with values > 0.

For the slope parameters, we specified normal distributions with mean zero, implying that they are theory-neutral with regard to potential effects. For the models including symbolic-number mapping, condition and their interaction as predictors, we ran prior sensitivity analyses, as Bayes factors can vary depending on the prior distribution; see Schad et al. (2023) or Nicenboim et al. (2021) for discussion. Specifically, we varied the standard deviation of the normal distribution, such that our prior assumptions were differently informed with regard to the expected effect sizes. Table 3 shows which priors were defined for each model and the resulting Bayes factor. In the main text, we report the results for Prior 1. As can be seen, for both experiments, the different priors affected the size of the Bayes factors. However, the general conclusions remained the same.

For the model that contained domain knowledge as the only fixed effect (Experiment 2), we assumed a normal distribution normal(0, 0.3) as the prior for the slope parameter.

**Table 3:** Prior sensitivity analyses for Experiments 1 and 2

	Predictors				
Models	Symbolic-number mapping	Condition	Symbolic-number mapping	Domain knowledge	Bayes factor
			$\times$ condition		
Experiment 1					
Model 1					
Prior 1	**normal(0, 0.5)**	—	—	—	83
Prior 2	**normal(0, 1)**	—	—	—	56
Prior 3	**normal(0, 2)**	—	—	—	28
Prior 4	**normal(0, 5)**	—	—	—	12
Experiment 2					
Model 1					
Prior 1	**normal(0, 0.5)**	—	—	—	34
Prior 2	**normal(0, 1)**	—	—	—	36
Prior 3	**normal(0, 2)**	—	—	—	19
Prior 4	**normal(0, 5)**	—	—	—	5
Experiment 2					
Model 2					
Prior 1	normal(0, 0.5)	normal(0, 2)	**normal(0, 0.5)**	—	0.62
Prior 2	normal(0, 0.5)	normal(0, 2)	**normal(0, 1)**	—	0.62
Prior 3	normal(0, 0.5)	normal(0, 2)	**normal(0, 2)**	—	0.37
Prior 4	normal(0, 0.5)	normal(0, 2)	**normal(0, 5)**	—	0.06
Experiment 2					
Model 3					
Prior 1	**normal(0, 0.5)**	—	—	normal(0, 0.3)	30
Prior 2	**normal(0, 1)**	—	—	normal(0, 0.3)	49
Prior 3	**normal(0, 2)**	—	—	normal(0, 0.3)	30
Prior 4	**normal(0, 5)**	—	—	normal(0, 0.3)	15

*Note.* Shown in bold is the effect of interest for which the sensitivity analyses were run

## Appendix C: Results with alternative measures of symbolic-number mapping

In addition to the analyses reported in the main text, we re-ran the analyses to ensure that the results also hold when using two alternative quantifications. The first is a common quantification in the symbolic-number mapping literature, the median absolute $\Delta$ (Eq. 3; Schneider et al., 2018), the second is a logarithmic measure that is more similar to our logarithmic measure of estimation accuracy, the median mapping OME (Eq. 4).^7^ Both are computed as deviation measures for the number indicated (i.e., estimate) from the number presented (i.e., actual) for each item *k* and each participant *j*:

3 $$\begin{aligned} Absolute \Delta _k{_j} = |{estimate_k{_j} - actual_k}|. \end{aligned}$$

4 $$\begin{aligned} Mapping OME_{kj} =|log_{10}\left( \frac{estimate_{kj}}{actual_k}\right) |. \end{aligned}$$

### Median absolute $\Delta$

For the slope parameter of symbolic-number mapping and its interaction with the number-line condition, we defined as prior a normal distribution normal(0, 0.00125) for the median absolute $$\Delta $$. Overall, the results closely mirrored the results presented in the main text. Estimation accuracy was associated with symbolic-number mapping in both Experiment 1 ($$b = 0.0017$$, $$CI_{95\%}$$ = [0.0002, 0.0032], BF_10_ $$= 7$$, standardized regression weight $$\beta = 0.05$$) and Experiment 2 ($$b = 0.0015$$, $$CI_{95\%}$$ = [0.0001, 0.0028], BF_10_ $$= 8$$, standardized regression weight $$\beta = 0.03$$). In addition, there was no interaction between symbolic-number mapping and number-line condition ($$b = -0.0003$$, $$CI_{95\%}$$ = [$$-0.0021, 0.0017$$], BF_10_ $$= 0.79 $$).

### Median Mapping OME

As priors, we used the priors defined in Prior 1 in Appendix B. Again, the results closely mirrored the results presented in the main text. Estimation accuracy was associated with symbolic-number mapping in both Experiment 1 ($b = 0.54$, $CI_{95\%}$ = [0.20, 0.89], BF_10_ $= 41$, standardized regression weight $\beta = 0.07$) and Experiment 2 ($b = 0.77$, $CI_{95\%}$ = [$-0.02, 1.57$], BF_10_ $= 5.00$, standardized regression weight $\beta = 0.02$). In addition, there was no interaction between symbolic-number mapping and number-line condition ($b = 0.16$, $CI_{95\%}$ = [$-0.72, 1.04$], BF_10_ $= 0.82$).

## Appendix D: Domain Engagement Question

The Domain Engagement Question was worded as follows (translated from German): “At the beginning of this study, you answered several questions about the populations of countries. How *frequently*, prior to this study, have you engaged with this topic (e.g., at school, at work, or in your leisure time)?”

The response options were: very rarely, rarely, rather rarely, neither rarely nor frequently, rather frequently, frequently, very frequently.

## Footnotes

1 Many real-world domains have highly skewed distributions spanning several orders of magnitude; for such distributions the OME is a better fit than more conventional indices such as the Pearson correlation coefficient or the mean deviation (Brown, 2002; Brown & Siegler, 1996; Groß et al., 2023). In such distributions, a single estimate can differ from others by several orders of magnitude, which would lead to an inflated impact of such outliers if more conventional indices were used. Using a log-based measure such as the OME minimizes the distorting effects of outliers, as under- and overestimation of the same order of magnitude is weighted equally (Brown, 2002). An OME of 1 indicates that the estimation is off by one order of magnitude (e.g., an estimated value of 10 million or 100,000 for an actual value of 1 million); an OME of 2 indicates that the estimation is off by two orders of magnitude (e.g., an estimated value of 100 million or 10,000 for an actual value of 1 million).

2 The BF is commonly interpreted as follows. A BF_10_ below 1/10 indicates strong evidence, between 1/10 and 1/3 moderate evidence, and between 1/3 and 1 weak evidence for *M*_0_. A BF_10_ larger than 10 indicates strong evidence, between 3 and 10 moderate evidence, and between 1 and 3 weak evidence for *M*_1_ (e.g., Jeffreys, 1998; Lee & Wagenmakers, 2014; van Doorn et al., 2023).

3 One might object that the association between the performances in the number-line task and the estimation task could be due to individual differences in how diligently participants engaged in solving the tasks. To address this possibility, we conducted additional analyses in which we controlled for how much time participants spent on each of the respective tasks. These analyses showed that the association between symbolic-number mapping and estimation accuracy was independent of the time spent on the tasks. The same held for the results of Experiment 2.

4 In Appendix C, we report the results for two alternative quantifications of symbolic-number mapping accuracy, namely, the median absolute $\Delta$ and the median mapping OME. The general pattern of results was the same for both quantifications of symbolic-number mapping.

5 In the preregistration, this research question was formulated as exploratory. Furthermore, our original formulation of the research question asked whether domain knowledge adds explanatory value to the prediction of estimation accuracy after accounting for symbolic-number mapping. When preparing the analyses, we realized that the other direction is theoretically more reasonable, as prior research has already shown that domain knowledge subserves real-world estimation.

6 As in Experiment 1, we also report the results for two alternative quantifications of symbolic-number mapping accuracy in Appendix C, namely the median absolute $\Delta$ and the median mapping OME. The general pattern of results was the same for both quantifications of symbolic-number mapping.
